# Supplementary material for: Tillage type and sentinel insect species affect the relative prevalence of the entomopathogenic fungus, Metarhizium robertsii, in soil
Source: PLoS One. 2025 Jan 13;20(1):e0317149. doi: 10.1371/journal.pone.0317149 (PMC11729919; doi:10.1371/journal.pone.0317149)
Supplement: S1 File — Management strategies for four organic feed grain and forage systems showing the cropping sequence for Entry 3 (Wheat-Corn-Soybean).S3 Table. Field operations by experimental system, rotation entry point, and date.S4 Table. Number of isolates by experimental system and crop of Metarhizium sp. from sentinel T. molitor and G. mellonella subjected to molecular analysis by sequencing the translation elongation factor-1 alpha (5α-TEF) by the methods of Kepler et al. [49] and described in Ahmad et al. [25] and Randhawa et al. [34]. All isolates were identified as M. robertsii. (DOCX) [file pone.0317149.s001.docx]

**Supplementary Materials**

**S Table 1 Three-year crop sequence in full entry cropping systems experiment Systems 1 – 4.**

|  |  | **Experimental Systems 1 - 3** | | |  | **Experimental System 4** | | |
| --- | --- | --- | --- | --- | --- | --- | --- | --- |
| **Cash Crop Year** |  | **Entry 1** | **Entry 2** | **Entry 3** |  | **Entry 1** | **Entry 2** | **Entry 3** |
| 2021 |  | Corn | Soybean | Wheat |  | Alfalfa (1 yr) | Wheat | Soybean |
| 2022 |  | Soybean | Wheat | Corn |  | Alfalfa (2 yr) | Alfalfa (1 yr) | Wheat |
| 2023 |  | Wheat | Corn | Soybean |  | Alfalfa (3 yr) | Alfalfa (2 yr) | Alfalfa (1 yr) |

**S Table 2. Management strategies for four organic feed grain and forage systems showing the cropping sequence for Entry 3 (Wheat-Corn-Soybean).**

|  |  | **System 1** | **System 2** | **System 3** | **System 4** |
| --- | --- | --- | --- | --- | --- |
| **Management strategy** | | Cash grain crops managed with inversion tillage, integrating winter cover crops using reduced tillage methods | Soybean phase of rotation managed with shallow tillage using a HSD, intended to reduce the intensity and depth of soil disturbance | Reduced tillage system intended to reduce disturbance to the extent possible | Perennial forage legume-grass mixture as a minimal soil disturbance baseline |
| **Year 1 (2021)** | **Winter** | Wheat planted using chisel tillage | Wheat planted with HSD | Wheat planted with HSD | Oat residue |
|  | **Spring** | Medium red clover no-till drilled into wheat | Wheat | Medium red clover no-till drilled into wheat | Alfalfa/orchardgrass mix planted with chisle plow |
|  | **Summer** | Wheat harvested | Wheat harvested | Red clover | Alfalfa/orchardgrass mix |
|  | **Fall** | Cereal rye no-till drilled into red clover | Oat/Austrian winter pea/forage radish cover crop mixture planted with HSD | Cereal rye no-till drilled into red clover | Alfalfa/orchardgrass mix |
| **Year 2 (2022)** | **Winter** | Cereal rye | Oat/Austrian winter pea/forage radish mixture | Cereal rye/red clover | Alfalfa/orchardgrass mix |
|  | **Spring** | Cereal rye/red clover terminated by inversion tillage | Cover crop terminated with HSD | Cereal rye/red clover terminated by inversion tillage | Alfalfa/orchardgrass mix |
|  | **Summer** | Corn planted Cover crop mixture interseeded into corn | Corn planted | Corn planted Cover crop mixture interseeded into corn | Alfalfa/orchardgrass mix |
|  | **Fall** | Corn harvested | Corn harvested Cereal rye planted with HSD | Corn harvested Cereal rye planted with HSD | Alfalfa/orchardgrass mix |
| **Year 3 (2023)** | **Winter** | Cover crop mixture | Cereal rye | Cereal rye | Alfalfa/orchardgrass mix |
|  | **Spring** | Cover crop mixture terminated with inversion tillage | Cereal rye terminated with HSD | Cereal rye terminated with roller crimper | Alfalfa/orchardgrass mix |
|  | **Summer** | Soybean planted | Soybean planted | Soybean no-till planted | Alfalfa/orchardgrass mix |
|  | **Fall** | Soybean harvested | Soybean harvested | Soybean harvested | Alfalfa/orchardgrass mix |

**S Table 3. Field operations by experimental system, rotation entry point, and date.**

| **Date** | **Entry** | **System 1** | | **System 2** | **System 3** | **System 4** | **Operation** |
| --- | --- | --- | --- | --- | --- | --- | --- |
| 17-Mar-2021 | 2 | x |  | | x |  | frost seed red clover |
| 5-Apr-2021 | 1 | x | x | | x | x | mow corn stubble |
| 5-Apr-2021 | 3 |  |  | |  | x | chisel plow, disc x 2, s-tine harrow |
| 7-Apr-2021 | 3 |  |  | |  | x | plant alfalfa, cultimulch |
| **16-Apr-2021** | **1,2,3** | **x** | **x** | | **x** | **x** | ***M. robertsii* sentinel assay** |
| 21-Apr-2021 | 1 |  | x | |  |  | mow cereal rye pre soybean |
| 23-Apr-2021 | 1 |  | x | |  |  | HSD 1x time, 2-3 inches |
| 27-Apr-2021 | 3 | x |  | | x |  | mow red clover |
| 27-Apr-2021 | 1 | x |  | |  | x | mow interseeded cover crop |
| 27-Apr-2021 | 3 | x |  | | x |  | moldboard plow red clover pre corn |
| 28-Apr-2021 | 1 | x |  | |  | x | moldboard plow interseeded cover crop |
| 3-May-2021 | 1 |  | x | |  |  | HSD cereal rye stubble |
| 6-May-2021 | 1 | x |  | |  | x | disc pre soybean |
| 6-May-2021 | 3 | x | x | | x |  | disc pre corn |
| 6-May-2021 | 3 |  | x | |  |  | mow cover crops, HSD pre corn |
| 12-May-2021 | 3 | x | x | | x |  | spread poultry litter |
| 14-May-2021 | 3 | x | x | | x |  | s-tine corn |
| 18-May-2021 | 3 | x | x | | x |  | cultimulch corn |
| 18-May-2021 | 1 | x | x | |  | x | s-tine, cultimulch soybean |
| 19-May-2021 | 3 | x | x | | x |  | plant corn, 35K/ac, 2.5'' depth |
| 24-May-2021 | 3 | x | x | | x |  | tine weed corn |
| 25-May-2021 | 1 |  |  | | x |  | roller crimp, plant no-till soybean |
| 27-May-2021 | 1 | x | x | |  | x | plant, cultimulch tilled soybean |
| 27-May-2021 | 3 | x | x | | x |  | tine weed corn |
| 2-Jun-2021 | 1 | x | x | |  | x | tine weed soybean |
| 7-Jun-2021 | 3 | x | x | | x |  | cultimulched previous corn |
| 7-Jun-2021 | 3 | x | x | | x |  | replant corn |
| 7-Jun-2021 | 3 | x | x | | x |  | s-tine to kill previous corn |
| 8-Jun-2021 | 1 | x | x | |  | x | tine weed beans, x 2 |
| 10-Jun-2021 | 3 | x | x | | x |  | tine weed corn |
| 18-Jun-2021 | 1 | x | x | |  | x | cultivate tilled beans |
| **21-Jun-2021** | **1,2,3** | **x** | **x** | | **x** |  | ***M. robertsii* sentinel assay** |
| 24-Jun-2021 | 3 | x | x | | x |  | cultivate corn |
| 24-Jun-2021 | 1 | x | x | |  | x | cultivate tilled beans |
| 28-Jun-2021 | 1 |  |  | | x |  | high residue cultivate no-till beans |
| 30-Jun-2021 | 3 |  |  | |  | x | mow, rake alfalfa |
| 2-Jul-2021 | 3 |  |  | |  | x | harvest alfalfa |
| 7-Jul-2021 | 3 | x | x | | x |  | cultivate corn |
| 7-Jul-2021 | 3 | x |  | |  |  | interseed 20, 8, 1 lb/ac ryegrass, crimson clover, radish |
| 21-Jul-2021 | 1 |  |  | | x |  | high residue cultivate no-till beans, x 2 |
| 21-Jul-2021 | 1 | x | x | |  | x | cultivate tilled soybeans, x 2 |
| 23-Jul-2021 | 2 | x | x | | x | x | harvest wheat |
| 13-Aug-2021 | 3 |  |  | |  | x | mow, rake alfalfa |
| 16-Aug-2021 | 2 |  | x | |  | x | mow post wheat |
| 24-Aug-2021 | 2 |  |  | |  | x | chisel plow, cultimulch, disc, s-tine post wheat |
| 25-Aug-2021 | 2 |  | x | |  |  | HSD post wheat |
| 7-Sep-2021 | 2 |  | x | |  | x | cultimulch post wheat |
| 8-Sep-2021 | 2 |  | x | |  |  | plant oat/pea/radish mix |
| 8-Sep-2021 | 2 |  |  | |  | x | plant oats |
| **17-Sep-2021** | **1,2,3** | **x** | **x** | | **x** | **x** | ***M. robertsii* sentinel assay** |
| 20-Sep-2021 | 2 | x |  | | x |  | harvest red clover |
| 7-Oct-2021 | 3 | x | x | | x |  | harvest corn |
| 8-Oct-2021 | 3 | x | x | | x |  | stalk chop corn residue |
| 11-Oct-2021 | 3 |  | x | | x |  | HSD after corn, 6-8'‘ depth |
| 11-Oct-2021 | 3 |  | x | | x |  | HSD after corn 2x, 4-5'' depth |
| 13-Oct-2021 | 3 |  | x | | x |  | plant cereal rye |
| 14-Oct-2021 | 2 | x |  | | x |  | drill cereal rye into clover, 60lb/ac |
| 20-Oct-2021 | 1 | x | x | | x | x | plot combine |
| 22-Oct-2021 | 1 | x |  | |  | x | chisel plow |
| 22-Oct-2021 | 1 | x | x | | x | x | cultimulch |
| 22-Oct-2021 | 1 |  | x | | x |  | HSD |
| 22-Oct-2021 | 1 | x | x | | x | x | apply manure, s-tine |
| 28-Oct-2021 | 1 | x | x | | x | x | plant wheat |
| 3-Nov-2021 | 3 |  |  | |  | x | mow, rake alfalfa |
| 3-Mar-2022 | 1 | x |  | | x |  | frost seed red clover |
| 13-Apr-2022 | 2 |  |  | |  | x | chisel plow, cultimulch, disc, s-tine, brillion seed alfalfa |
| **25-Apr-2022** | **1,2,3** | **x** | **x** | | **x** | **x** | ***M. robertsii* sentinel assay** |
| 25-Apr-2022 | 2 | x |  | | x |  | mow clover/rye pre corn |
| 28-Apr-2022 | 2 | x | x | | x |  | apply poultry litter pre corn |
| 29-Apr-2022 | 3 |  | x | |  |  | HSD, 3'', pre soybean |
| 29-Apr-2022 | 3 | x | x | |  |  | mow cereal rye and interseed cover crop pre soybean |
| 29-Apr-2022 | 2 | x |  | | x |  | moldboard plow 9-10'' depth, pre corn |
| 29-Apr-2022 | 3 | x |  | |  |  | moldboard plow 9-10'' depth, pre soybean |
| 10-May-2022 | 2 |  | x | |  |  | HSD |
| 12-May-2022 | 2 | x | x | | x |  | disc pre corn |
| 12-May-2022 | 3 | x | x | |  |  | disc pre soy |
| 12-May-2022 | 2 | x | x | | x |  | s-tine |
| 12-May-2022 | 3 | x | x | |  |  | s-tine |
| 13-May-2022 | 2 | x | x | | x |  | cultimulch |
| 13-May-2022 | 3 | x | x | |  |  | cultimulch |
| 24-May-2022 | 3 |  |  | |  | x | harvest alfalfa |
| 25-May-2022 | 3 |  |  | | x |  | plant no-till soybean |
| 25-May-2022 | 3 |  |  | |  | x | bale alfalfa |
| 25-May-2022 | 3 |  |  | | x |  | roller crimp no-till soybean |
| 31-May-2022 | 3 | x | x | |  |  | plant soybean |
| 31-May-2022 | 2 | x | x | | x |  | plant corn |
| 31-May-2022 | 3 | x | x | |  |  | cultimulch |
| 31-May-2022 | 2 | x | x | | x |  | cultimulch |
| 3-Jun-2022 | 3 | x | x | |  |  | tine weed soybean |
| 3-Jun-2022 | 2 | x | x | | x |  | tine weed corn |
| 10-Jun-2022 | 2 | x | x | | x |  | rotary hoe corn |
| 10-Jun-2022 | 3 | x | x | |  |  | rotary hoe soybean |
| 16-Jun-2022 | 2 | x | x | | x |  | rotary hoe corn |
| 16-Jun-2022 | 3 | x | x | |  |  | rotary hoe soybean |
| **20-Jun-2022** | **1,2,3** | **x** | **x** | | **x** |  | ***M. robertsii* sentinel assay** |
| 20-Jun-2022 | 2 | x | x | | x |  | cultivate corn |
| 20-Jun-2022 | 3 | x | x | |  |  | cultivate soybean |
| 28-Jun-2022 | 3 | x | x | |  |  | cultivated soybean |
| 28-Jun-2022 | 2 | x | x | | x |  | cultivated corn |
| 5-Jul-2022 | 2 | x |  | |  |  | interseed ryegrass, crimson clover, radish |
| 5-Jul-2022 | 3 |  |  | | x |  | high residue cultivate no-till soybean |
| 8-Jul-2022 | 2, 3 |  |  | |  | x | harvest alfalfa |
| 8-Jul-2022 | 2,3 |  |  | |  | x | rake 1- and 2-year alfalfa |
| 8-Jul-2022 | 3 | x | x | |  |  | cultivate soybean |
| 11-Jul-2022 | 2,3 |  |  | |  | x | rake alfalfa |
| 13-Jul-2022 | 3 |  |  | | x |  | high residue cultivate no-till soybean |
| 20-Jul-2022 | 1 | x | x | | x | x | harvest wheat |
| 11-Aug-2022 | 3 |  |  | |  | x | harvest 2-year alfalfa |
| 16-Aug-2022 | 3 |  |  | |  | x | rake, bale 2-year alfalfa |
| 16-Aug-2022 | 1 |  | x | |  | x | mowed wheat stubble |
| 17-Aug-2022 | 2 |  |  | |  | x | mow weeds above alfalfa |
| 17-Aug-2022 | 1 |  |  | |  | x | chisel plow, cultimulch, disc post wheat |
| 17-Aug-2022 | 1 |  | x | |  |  | HSD post wheat |
| 17-Aug-2022 | 1 |  |  | |  | x | s-tine |
| 10-Sep-2022 | 1 |  | x | |  | x | cultimulch |
| 10-Sep-2022 | 1 |  | x | |  |  | plant oat/pea/radish cover crop |
| 10-Sep-2022 | 1 |  |  | |  | x | plant oats |
| 13-Sep-2022 | 1 | x |  | | x |  | mow clover |
| 13-Sep-2022 | 2 |  |  | |  | x | mow alfalfa |
| 16-Sep-2022 | 1 | x |  | | x |  | bale clover |
| 16-Sep-2022 | 2 |  |  | |  | x | bale alfalfa |
| **23-Sep-2022** | **1,2,3** | **x** | **x** | | **x** | **x** | ***M. robertsii* sentinel assay** |
| 6-Oct-2022 | 3 |  |  | |  | x | rake, bale alfalfa |
| 7-Oct-2022 | 2 | x | x | | x |  | harvest corn |
| 12-Oct-2022 | 2 |  |  | | x |  | Seed cereal rye post corn, 3 bu/ac |
| 12-Oct-2022 | 2 |  | x | |  |  | Seed cereal rye post corn, 3 bu/ac |
| 12-Oct-2022 | 1 | x |  | | x |  | Seed cereal rye into clover |
| 17-Oct-2022 | 3 | x | x | | x |  | harvest soybeans, apply manure |
| 18-Oct-2022 | 3 | x |  | |  |  | chisel plow, cultimulch, disc post soybean |
| 18-Oct-2022 | 3 |  | x | | x |  | cultimulch, HSD post soybean |
| 18-Oct-2022 | 3 | x | x | | x |  | s-tine, plant wheat |
| 21-Feb-2023 | 3 | x |  | | x |  | frost seeded medium red clover |
| 4-Apr-2023 | 1 |  |  | |  | x | chisel plow, heavy disc, s-tine, cultimulch |
| **10-Apr-2023** | **1,2,3** | **x** | **x** | | **x** | **x** | ***M. robertsii* sentinel assay** |
| 10-Apr-2023 | 1 |  |  | |  | x | plant, cultimulch alfalfa |
| 10-May-2023 | 1 | x | x | | x |  | mow CC |
| 10-May-2023 | 2 | x | x | |  |  | mow CC |
| 10-May-2023 | 1 | x |  | | x |  | moldboard plow |
| 10-May-2023 | 2 | x |  | |  |  | moldboard plow |
| 11-May-2023 | 1 |  | x | |  |  | high speed disc 2x |
| 11-May-2023 | 2 |  | x | |  |  | high speed disc 2x |
| 11-May-2023 | 1 | x | x | | x |  | heavy disc |
| 11-May-2023 | 2 | x | x | |  |  | heavy disc |
| 11-May-2023 | 1 | x | x | | x |  | s-tine |
| 11-May-2023 | 2 | x | x | |  |  | s-tine |
| 12-May-2023 | 1 | x | x | | x |  | cultimulch |
| 12-May-2023 | 2 | x | x | |  |  | cultimulch |
| 15-May-2023 | 1 | x | x | | x |  | apply manure |
| 24-May-2023 | 2 |  |  | | x |  | roller crimp, plant no-till soybeans |
| 25-May-2023 | 2,3 |  |  | |  | x | mow, rake alfalfa off plot |
| 7-Jun-2023 | 1 | x | x | | x |  | cultimulch, plant corn |
| 7-Jun-2023 | 2 | x | x | |  |  | cultimulch, plant soybean |
| 10-Jun-2023 | 1 | x | x | | x |  | tine weed |
| 10-Jun-2023 | 2 | x | x | |  |  | tine weed |
| 18-Jun-2023 | 1 | x | x | | x |  | rotary hoe |
| 18-Jun-2023 | 1,2 |  | x | |  |  | cultivate |
| 20-Jun-2023 | 1 | x | x | | x |  | cultivate |
| 20-Jun-2023 | 2 | x | x | |  |  | rotary hoe, cultivate |
| **29-Jun-2023** | **1,2,3** | **x** | **x** | | **x** |  | ***M. robertsii* sentinel assay** |
| 30-Jun-2023 | 1 | x | x | | x |  | cultivate corn |
| 30-Jun-2023 | 2 | x | x | |  |  | cultivate soybean |
| 6-Jul-2023 | 1 | x | x | | x |  | cultivate corn |
| 6-Jul-2023 | 2 | x | x | |  |  | cultivate soybean |
| 6-Jul-2023 | 1,2,3 |  |  | |  | x | alfalfa yield strip |
| 6-Jul-2023 | 1,2,3 |  |  | |  | x | mow alfalfa |
| 10-Jul-2023 | 1,2,3 |  |  | |  | x | bale alfalfa |
| 11-Jul-2023 | 2 |  |  | | x |  | high residue cultivate no-till beans |
| 13-Jul-2023 | 1 | x | x | | x |  | cultivate corn |
| 13-Jul-2023 | 2 | x | x | |  |  | cultivate soybean |
| 19-Jul-2023 | 1 | x |  | |  |  | interseeded |
| 19-Jul-2023 | 2 | x | x | |  |  | cultivate soybean |
| 26-Jul-2023 | 3 | x | x | | x |  | harvested wheat |
| 1-Aug-2023 | 2 |  |  | | x |  | high residue cultivate |
| 17-Aug-2023 | 1,2,3 |  |  | |  | x | alfalfa yield strip |
| 17-Aug-2023 | 1,2,3 |  |  | |  | x | mow alfalfa |
| 17-Aug-2023 | 3 |  | x | |  |  | mow weeds |
| 17-Aug-2023 | 3 | x |  | | x |  | mow weeds above clover |
| 22-Aug-2023 | 1,2,3 |  |  | |  | x | rake alfalfa off plot |
| 5-Sep-2023 | 3 |  | x | |  |  | HSD, cultimulch |
| 8-Sep-2023 | 3 |  | x | |  |  | cultimulch |
| 8-Sep-2023 | 3 |  | x | |  |  | plant oat/pea/radish |
| 12-Sep-2023 | 3 | x |  | | x |  | mow weeds above clover |
| **15-Sep-2023** | **1.2.3** | **x** | **x** | | **x** | **x** | ***M. robertsii* sentinel assay** |
| 29-Sep-2023 | 1,2,3 |  |  | |  | x | mow alfalfa |
| 4-Oct-2023 | 1,2,3 |  |  | |  | x | raked alfalfa off plot |
| 12-Oct-2023 | 3 | x |  | | x |  | drillcereal rye into clover |
| 25-Oct-2023 | 1 | x | x | | x |  | harvest corn |
| 25-Oct-2023 | 1 |  | x | | x |  | mow corn stalks |
| 26-Oct-2023 | 1 |  | x | | x |  | HSD x2, drill 3 bu/ac cereal rye, |
| 6-Nov-2023 | 2 | x | x | | x |  | harvested soybean |
| 6-Nov-2023 | 2 | x |  | |  |  | chisel plow, disc, cultimulch |
| 7-Nov-2023 | 2 |  | x | | x |  | HSD |
| 7-Nov-2023 | 2 | x | x | | x |  | plant wheat |

**S Table 4**. **Number of isolates by experimental system and crop of *Metarhizium* sp. from sentinel *T. molitor* and *G. mellonella* subjected to molecular analysis by sequencing the translation elongation factor-1 alpha (5α-TEF) by the methods of Kepler et al. [49] and described in Ahmad et al. [25] and Randhawa et al. [34]. All isolates were identified as *M. robertsii*.**

| **System** | **Crop** | ***T. molitor*** | ***G. mellonella*** |
| --- | --- | --- | --- |
| **System 1** | Corn | 6 | 11 |
|  | Cereal Rye | 6 | 3 |
|  | Red Clover/Annual Ryegrass | 5 | 1 |
|  | Wheat | 9 | 8 |
|  | Soybean | 7 | 1 |
| **System 2** | Corn | 7 | 3 |
|  | Cereal Rye | 8 | 4 |
|  | Red Clover/Annual Ryegrass | 8 | 6 |
|  | Wheat | 9 | 7 |
|  | Soybean | 10 | 2 |
| **System 3** | Corn | 8 | 7 |
|  | Cereal Rye | 6 | 6 |
|  | Red Clover/Annual Ryegrass | 0 | 4 |
|  | Wheat | 4 | 4 |
|  | Soybean | 9 | 3 |
| **System 4** | Corn | 5 | 0 |
|  | Cereal Rye | 8 | 5 |
|  | Red Clover/Annual Ryegrass | 5 | 2 |
|  | Wheat | 5 | 6 |
|  | Soybean | 8 | 1 |
|  | Oat | 1 | 0 |
|  | Alfalfa/Orchardgrass | 0 | 4 |
| **Total** |  | **134** | **88** |
